# Effect of Knee Joint Meniscus Tears on Joint Cartilage Contact and Pressure with Finite Element Analysis

**DOI:** 10.3390/biomedicines14040869

**Published:** 2026-04-10

**Authors:** Cengizhan Kurt, Arif Gök

**Affiliations:** 1 Department of Orthopaedics and Traumatology, Faculty of Medicine, Izmir Bakircay University, 35665 İzmir, Turkey; cengizhan.kurt@bakircay.edu.tr; 2 Department of Industrial Design, Faculty of Architecture, Kutahya Dumlupinar University, 43100 Kutahya, Turkey

**Keywords:** menisci tear, finite element analysis, stress distribution, radial tear, joint biomechanics

## Abstract

**Background/Objectives:** The medial meniscus is crucial for load transmission and knee stability. Meniscal tears disrupt joint biomechanics, increasing the risk of cartilage degeneration. However, few studies have quantitatively compared how different tear types affect stress and contact mechanics using finite element analysis (FEA). This study aims to analyze stress distributions for various meniscal tear types and develop a predictive model for meniscal stress behavior. This study investigates how stress distributions differ between healthy and torn medial menisci under identical loading conditions. The study examines which meniscal tear type produces the highest stress concentrations. The effects of different tear types on penetration, gap formation, pressure distribution, and sliding distance at the meniscus interface are also analysed. **Materials and Methods:** The FEA model of the knee joint, including femoral and tibial cartilage and the medial meniscus, was developed. Simulations were conducted for a healthy meniscus and for menisci with radial, horizontal and complex tears. Stress, penetration, gap, pressure, and sliding distance were calculated, and a mathematical model describing their relationships was established. **Results:** All torn menisci exhibited significantly higher stresses than the healthy meniscus (*p* < 0.001). Radial tears generated the highest stress concentrations (*p* < 0.001). Pressure was mainly influenced by meniscal geometry, while the gap remained nearly constant. Penetration increased slightly (*p* < 0.05). The predictive model demonstrated a strong correlation between meniscal stress and interface parameters (R^2^ > 0.9). In a healthy meniscus, stress distribution is homogeneous (≈26 MPa). Stress concentration increases depending on the tear type: limited in a horizontal tear (≈26.5 MPa), significant in a vertical tear (≈30.8 MPa), and highest in a radial tear (≈40.6 MPa). These results indicate that as the tear progresses, the load-bearing capacity of the meniscus decreases, and stresses concentrate at the tear edges. **Conclusions:** Meniscal tears, especially radial ones, substantially alter knee biomechanics and elevate tissue stress. These biomechanical insights highlight the importance of early diagnosis and targeted rehabilitation strategies to prevent further cartilage damage and osteoarthritis progression.

## 1. Introduction

Menisci are circular, wedge-shaped fibrocartilaginous constructs found between the tibial plateau and the femoral condyles. Menisci serve significant purposes in the knee joint. Menisci also help to stabilize the knee joint in addition to load bearing, load transfer, shock absorption, and resistance to impact. Menisci are circular, wedge-shaped fibrocartilaginous structures located between the femoral condyles and the tibial plateau [1]. Menisci have important functions in the knee joint. In addition to load bearing, load transfer, shock absorption, and resistance to impact, menisci also contribute to stabilization of the knee joint. The incongruity of the knee joint, with its rounded femoral surface and flat tibial surface, would lead to joint incongruity. The menisci, fibrocartilaginous structures mitigate this incongruity. The menisci’s crescent shape and wedge-like form help distribute compressive forces to the articular cartilage in a concentric manner, through the menisci. Circumferential stresses are also transmitted to the meniscal root via the meniscal peripheral fibers. This load distribution has a protective effect on the articular cartilage and prevents degeneration [1,2]. In the presence of a healthy meniscus, the joint contact area increases 2.5 times. This increased contact area reduces the contact stress between the bones. In the presence of meniscal damage, the contact area will decrease and stress will increase. If this altered loading environment persists, the resulting increase in contact stress primarily induces degenerative changes in the articular cartilage and adaptive remodeling of the underlying subchondral bone, ultimately contributing to the development of osteoarthritis. Total meniscectomy has been shown to reduce the volume of the tibiofemoral contact area by 50–70%, exposing the articular cartilage to high pressure, while recent studies have shown that even much smaller meniscal volume losses can disrupt knee mechanics, leading to increased joint pressure [2]. The concave upper surface and flat lower surface of the meniscus contribute to joint stability by facilitating joint congruity and working with the meniscotibial and meniscofemoral ligaments [3]. Since the medial meniscus is connected to the medial collateral ligament, the lateral meniscus is more mobile compared to the medial meniscus. Meniscal tears can be classified according to various characteristics such as tear location, tear pattern, and time of injury occurrence [4].

Recent research using finite element analysis has shed light on the biomechanical effects of meniscal injuries and the mechanics of cartilage contact in the knee joint. For example, Mao et al. [5] showed that the distribution of load in the knee joint is significantly altered by the location of medial meniscus tears. Similarly, stress concentration around horizontal meniscal tears during knee flexion was noted by Yan et al. [6], which may aid in the spread of tears. Additionally, research by Ma et al. [7] and Deng et al. [8] demonstrated that meniscal extrusion or meniscectomy can significantly raise cartilage contact stresses, which may hasten the development of osteoarthritis. Chen et al. [9] showed the stress distribution in the knee joint in cases of horizontal meniscus tears and partial meniscectomy was examined using FEA at different flexion angles. The study shows that meniscectomy can increase cartilage contact stress in the joint. Yu et al. [10] highlighted that finite element modeling is an important tool for clinical decision-making and surgical planning in the evaluation of meniscal tears.

Many studies have suggested that the high shear and tensile stresses occurring in the articular cartilage following meniscectomy may contribute to cartilage degeneration and the development of osteoarthritis [11]. Previous research has investigated different biomechanical and structural aspects of the meniscus. For example, Kim et al. [12]. evaluated the effect of posterior root repair of the medial meniscus on tibiofemoral contact mechanics and examined the impact of meniscal allograft transplantation (MAT) combined with medial collateral ligament (MCL) release at different flexion angles. Similarly, Danso et al. [13]. analyzed the composition and structural organization of the human knee meniscus, including proteoglycan content, collagen content, and collagen fibril orientation, in a site- and depth-dependent manner to better understand structure–function relationships. In another study, Seyfi et al. [14]. developed a nonlinear poroviscoelastic finite element model to characterize the mechanical behavior of meniscal tissue based on its microstructural properties. Despite these advances, biomechanical studies on the human meniscus still show considerable discrepancies in reported results, sometimes differing by up to an order of magnitude due to variations in testing protocols and sample characteristics. This variability highlights the need for standardized testing approaches and controlled biomechanical analyses when evaluating meniscal mechanics and implant designs [15].

Improving medical implant designs is a unique challenge for engineers and clinicians due to the difficulty of realistic testing. Unlike external products, implants undergo validation in life-like scenarios, including computer models, experimental tests, and clinical trials [16]. Nowadays, computer-aided FEA and computational fluid dynamics (CFD) are employed to address various processes, including metal turning, bone drilling, bone screwing, water jet processes, and erosion-corrosion phenomena. These technologies are also utilized in studying the fatigue behavior of implant materials, simulating COVID-19 and other infections, and optimizing the configurations of implant materials [17,18]. The studies by Senalp et al., Kayabasi et al., and Gok and Inal all used FEA to investigate the behavior of various designs and materials in the context of hip prostheses and femoral neck fractures. Senalp et al. [19] used FEA to model and evaluate four different stem shapes for hip prostheses, and analyzed their static, dynamic, and fatigue behavior. Kayabasi et al. [20] also used FEA to investigate the static, dynamic, and fatigue behaviors of the implants. In their study, Gok and Inal [18] employed FEA to explore the optimal stable fixation approach, testing five different screw configuration types in a femoral neck fracture model. Atmaca vd. [21] aimed to investigate how the location and extent of medial meniscectomy affect the loading on the tibial articular cartilage. We used FEA on solid models to compare loading in knees after different types of meniscectomy to loading in healthy knees.

The primary objective of this study is to investigate in detail the effects of different types of meniscal tears on knee joint biomechanics using FEA. Specifically, by determining the changes in stress, pressure, gap, and penetration values caused by tears, it is aimed to better understand the risk of damage to the meniscal tissue and the interactions between the joint surfaces. It is hoped that the findings will contribute to the development of methods used in the diagnosis and treatment of meniscal tears.

Recent advancements in biomaterials and joint microenvironment modulation have shown promising therapeutic potential in managing osteoarthritis and tendon pathologies. For example, engineered porous PLGA microspheres incorporating metal-phenolic nanomedicine have been demonstrated to neutralize acidic microenvironments, inhibit inflammatory responses, and preserve cartilage homeostasis in osteoarthritic models, highlighting a multifaceted strategy for joint degeneration therapy [22]. Additionally, precision characterization of tendinopathy has revealed distinct molecular subtypes—including hypoxic atrophic and inflammatory proliferative classes which exhibit divergent pathogenic mechanisms and suggest tailored therapeutic interventions for tendon disorders [23]. Furthermore, efforts to reinforce biomaterial matrices with bioactive nanoparticles such as zinc oxide and hydroxyapatite have enhanced mechanical properties and biological responses, underscoring the role of composite nanostructures in orthopedic regenerative applications [24]. These collective findings support a more comprehensive integration of microenvironment-modulating biomaterials and subtype-specific tendon pathology frameworks in the present study.

The thorough finite element analysis of various meniscal tear types under identical loading conditions to assess their biomechanical effects on the knee joint is what makes this study novel. The current study concurrently examines stress distribution, pressure, gap formation, penetration, and sliding distance at the meniscus–cartilage interface, in contrast to earlier studies that typically concentrate on a single tear configuration or constrained biomechanical parameters.

Additionally, the study offers a comparative analysis of various tear types, enabling a better comprehension of how various tear morphologies affect joint biomechanics and possible tissue damage. The study provides a more comprehensive evaluation of meniscal tear mechanics by analyzing these biomechanical parameters collectively within the same modeling framework.

This method offers fresh perspectives on the mechanical behavior of torn menisci and could contribute to better biomechanical knowledge for meniscal injury diagnosis and treatment planning.

## 2. Materials and Methods

The material properties adopted in the present finite element model were obtained from previously published studies and are summarized in Table 1, together with their corresponding literature sources. Modelling processes used Reverse Engineering (RE) techniques and advanced modelling techniques.

### 2.1. Computer-Aided Modelling Process

The computer analysis in this study was based on a physical knee model often employed in medical education and surgical training. This paradigm does not make use of live or cadaveric specimens and does not come from human tissue. The computational knee model was created by reconstructing the scanned geometry after digitizing the physical model using a three-FEA element analysis (ANSYS Workbench). Ethical approval was not necessary since no biological materials or human subjects were used. It should also be noted that the primary goal of this research was not to replicate patient-specific anatomy, but rather to utilize finite element analysis to examine the biomechanical consequences of various meniscal tear configurations under constant geometric circumstances.

**Figure 1:**
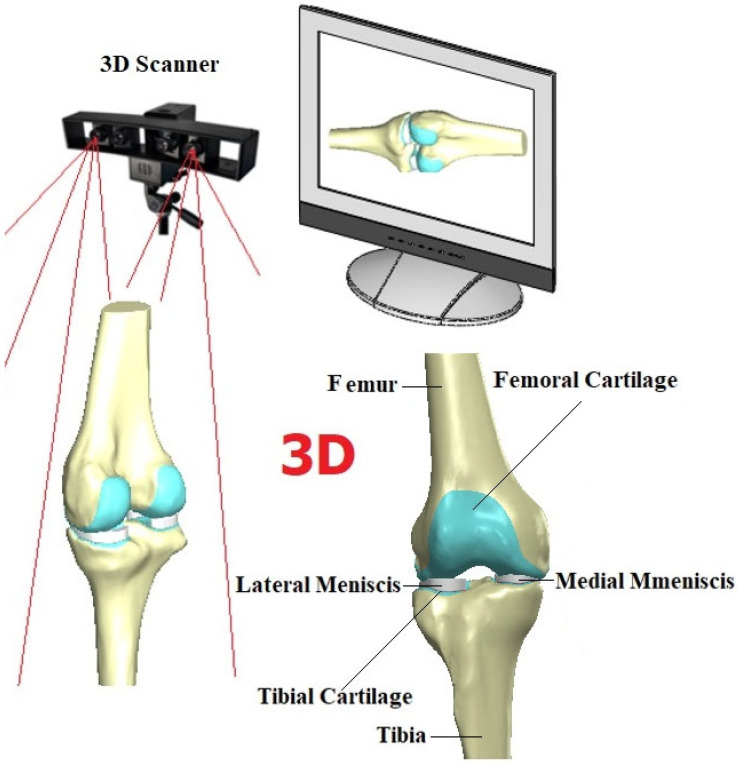
RE process for knee model and 3D of the knee model.

**Figure 2:**
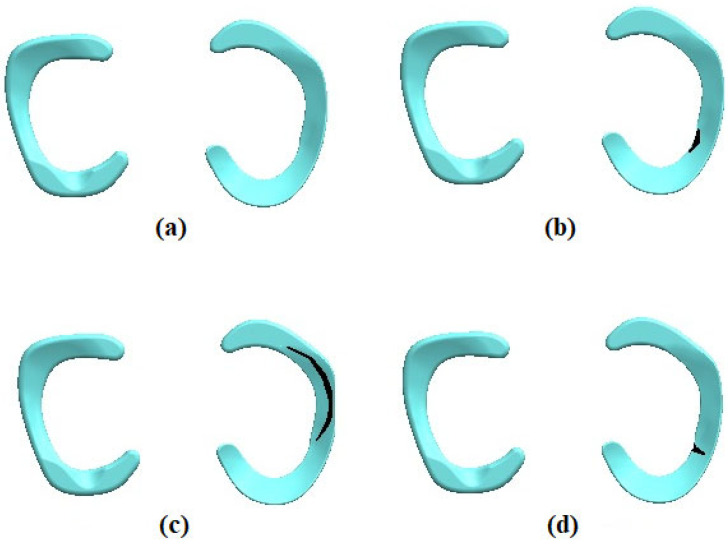
The healthy meniscus and tear meniscus models, (**a**) Healthy meniscus, (**b**) horizontal meniscus tear, (**c**) longitudinal (vertical) meniscus tear, (**d**) radial meniscus tear.

A 3D scanner (Shining 3D, Hangzhou, China) was used to obtain a point cloud of the human femur and tibia model which has a knee model during the scanning process via Reverse Engineering (RE) in Figure 1. RE involves employing specialized software and hardware to replicate intricate shapes and designs. This approach streamlines the product design process. Its significance is particularly pronounced when Computer-Aided Design (CAD) (SolidWorks) models of products are no longer accessible. Deviating from the conventional production sequence, reverse engineering commences with the measurement of an existing object. This measurement data is then utilized to generate a 3D model, enabling the leveraging of the benefits associated with Computer-Aided Design (CAD) technologies [25]. Using the Geomagic Studio program (Geomagic for Solidworks), the obtained point cloud was transformed into a 3D femur model and screw, facilitating further analysis and evaluation (Table 1). The femur and tibia were modeled using SolidWorks (2018). After that using advanced modelling techniques, meniscus and cartilage were obtained in SolidWorks platform as seen in Figure 1 both healthy meniscus and tears models in Figure 2.

**Table 1:** The mechanical properties of bone and soft tissues [26].

Material	Young Modulus (MPa)	Poisson Ratio
Femur	17,000	0.3
Tibia	14,000	0.3
Cartilage	5	0.46
Menisci	59	0.49

In this study, a numerical knee model was developed using anatomically accurate geometry derived from structured light 3D scanning of a real knee model. The scanned data was transformed into a comprehensive three-dimensional CAD model, which was used as the foundation for finite element simulations. The purpose of the research was to assess the biomechanical consequences of widely acknowledged meniscal tear types (radial, horizontal, and longitudinal) in a regulated and replicable manner, rather than to replicate patient-specific traumatic injury. To guarantee clinical relevance, tear patterns were created in consultation with a seasoned orthopedic surgeon. Although the model makes some assumptions, such as homogeneous material qualities and straightforward loading circumstances, these do not negate the biomechanical significance of the findings but rather set the limits of interpretation. This framework enables a comparative examination of how various meniscal tear patterns affect joint behavior and stress distribution mechanically.

Anatomical geometry was gotten by scanning a physical knee model frequently used for medical education and training. With a nominal scanning resolution of around 0.1 mm, the model was digitized using an Einscan Pro structured-light 3D (Shining 3D, Hangzhou, China) scanner with sub-millimeter spatial resolution. To guarantee full surface coverage, several scans were taken from various orientations. The resulting point clouds were aligned and merged using the scanner’s proprietary software (Geomagic for Solidworks) and a triangulated surface mesh was generated for subsequent geometric processing and FEA. Ethical clearance was not necessary since no human participants or biological tissues were employed.

As seen in Figure 2, using a three-dimensional modeling technique created in close cooperation with a seasoned orthopedic surgeon, the meniscal tear models were made. Based on clinically pertinent meniscal tear patterns, the tear morphology, location, and orientation were characterized. Rather than via damage or material degradation models, meniscal tears were shown in the numerical model as geometric discontinuities introduced straight into the three-dimensional meniscus geometry. This technique lets the tear be clearly shown as physical separation inside the structure. To clarify Section 2, more details describing this modeling approach have been included.

### 2.2. Loading and Boundary Conditions

**Figure 3:**
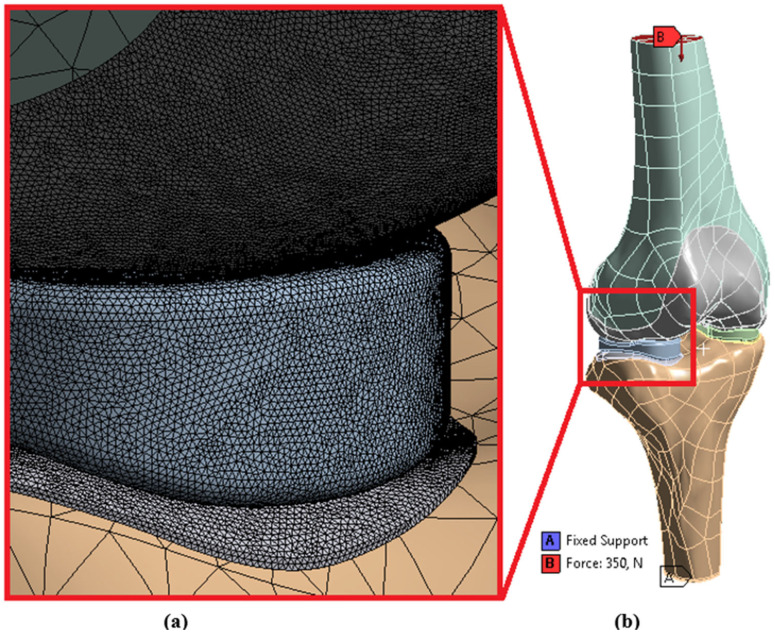
(**a**) Mesh, (**b**) Loading and boundary conditions.

The FEA modeling was conducted using tetrahedron elements in Ansys Workbench (2019), with four different 3D models imported (Figure 3). The model consisted of 114,988 nodes and 60,922 elements. While a mesh size of 2 mm is chosen for femur and tibia, the size for cartilages and menisci is selected to be 0.25 mm.

As reported in earlier studies [26,27], the applied compressive load of 350 N was selected to represent normal working conditions of the knee joint in a simplified and controlled manner, directed axially toward the femoral head while the tibia was fully constrained to ensure fixation. The 350 N load was deemed suitable for our comparative analysis of various meniscal tear types, allowing clear evaluation of stress, pressure, gap, and penetration changes without introducing excessive nonlinearity or computational complexity, despite the fact that higher loads, such as 800 N, have been applied in other studies to reflect full extension forces during the gait cycle [19]. This load selection preserves the stability and interpretability of the model while offering a physiologically reasonable baseline [28].

A single knee model was studied under a fixed loading circumstance to minimize variation and highlight basic biomechanical reactions. For computational simplicity, the meniscus was modeled as a homogeneous material; the constraints connected with simplified loading scenarios and the absence of anisotropic, region-dependent meniscal properties are recognized, which could compromise the generalizability of the results.

Bonded contact types were defined between bone and cartilages, and frictionless contact types were defined between menisci and cartilages [21]. In this study, only computer-aided numerical analysis solutions were conducted. The properties of the materials were sourced from the literature. The analyses were repeated three times to ensure reliability.

**Figure 4:**
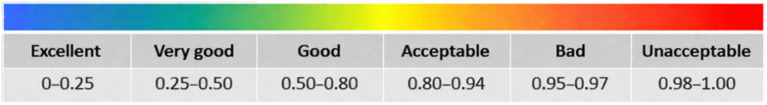
Skewness mesh metrics spectrum.

**Figure 5:**
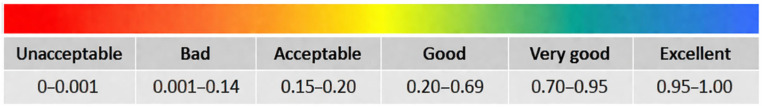
Orthogonal quality mesh metrics spectrum.

According to Mesh Metric, developed by Ansys, Inc. [29], mesh quality is very important in terms of reliability of the results. Low Orthogonal Quality or high skewness values are not recommended. Generally, try to keep minimum orthogonal quality > 0.15 or maximum skewness < 0.95. However, these values may be different depending on physics and the location of the cell. Fluent reports negative cell volumes if the mesh contains degenerate cells [29]. Skewness mesh metrics spectrum and Orthogonal Quality mesh metrics spectrum are given in Figure 4 and Figure 5.

**Figure 6:**
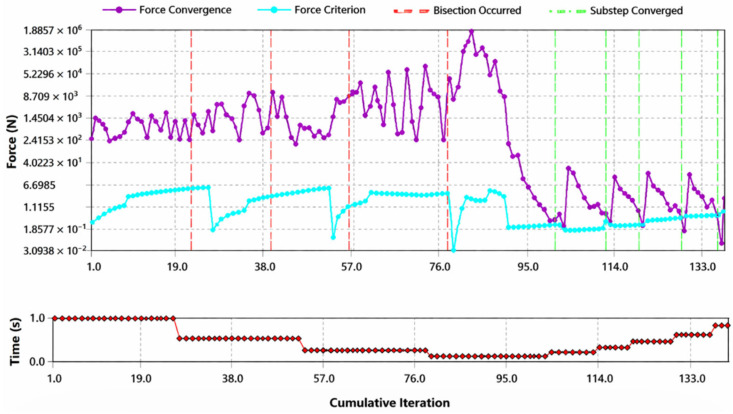
The convergence analysis.

In our analysis results, average skewness value was calculated as 0.62. According to Figure 4, this value is good (0.50–0.80). According to Figure 5, this value is good (0.20–0.69). The convergence analysis is given in Figure 6.

## 3. Results

This study, conducted using FEA, investigated the effects of different types of meniscal tears on knee joint biomechanics (Table 2). Analyses of healthy menisci and menisci with various types of tears revealed that tears increase stress within the meniscus, leading to tissue damage and disrupting joint stability, which accelerates cartilage damage. This situation can pave the way for joint diseases such as osteoarthritis in the long term. The findings emphasize the importance of early diagnosis and treatment of meniscal tears and provide a significant foundation for future studies.

According to the values in Table 2, the average stress experienced by a healthy meniscus under normal working conditions was determined to be 26.145 MPa. However, this value increased significantly in torn menisci; for example, in the case of a radial tear, the stress increased up to 40.616 MPa. This increase indicates that excessive stress tears the meniscal tissue, increasing the risk of damage. The average pressure exerted by a healthy meniscus on the joint surface is 14.806 Pa, and this pressure value generally does not change significantly in torn menisci. This suggests that the pressure value is more dependent on the geometric properties of the joint surfaces. The gap value is negative in all cases, indicating tight contact between the joint surfaces, and it was observed that tears did not significantly affect this value. The negative gap values indicate that the surfaces are in complete contact with each other in the biomechanical model and that soft tissue penetration (compression) occurs under load. This is proof that the physical interaction required for the generation of contact pressure is successfully achieved in the FEA model.

When looking at the penetration value, while this value is 0.0080715 mm in a healthy meniscus, tears, especially radial tears, increase this value somewhat. This indicates that tears can increase friction between the joint surfaces, leading to cartilage damage in the long term. Finally, the sliding distance value, which represents the sliding distance of the surfaces relative to each other during joint movements, showed small differences between tear types, but it was generally observed that this distance was not significantly affected.

Pressure is the normal contact force per unit area acting between contacting surfaces. Gap is the normal distance between contacting surfaces. Penetration is the numerical interpenetration of contacting surfaces within the contact algorithm, not representing physical overlap. Sliding distance is the cumulative relative tangential displacement between contacting surfaces.

**Figure 7:**
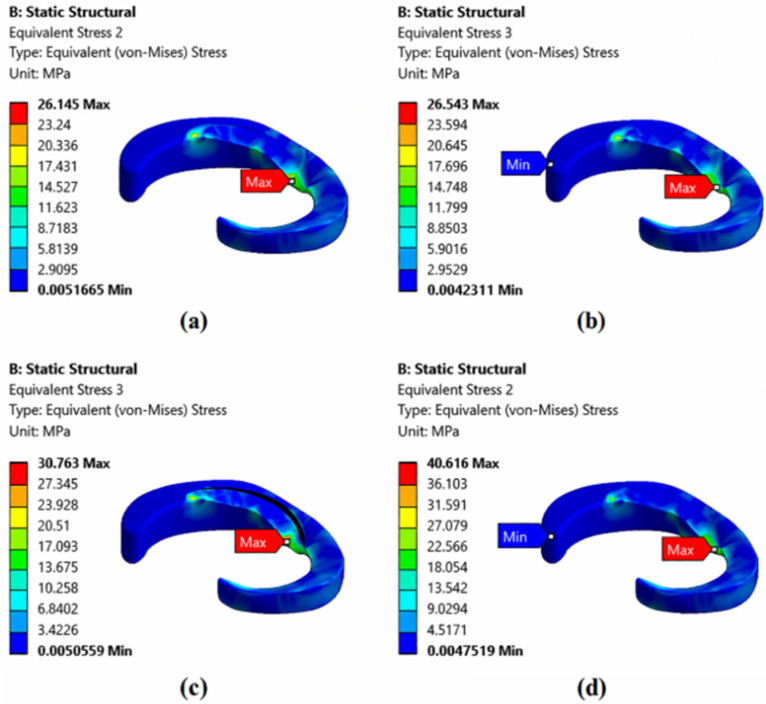
The stresses occurring at the medial meniscus for (**a**) healthy meniscus, (**b**) horizontal meniscus tear, (**c**) longitudinal (vertical) meniscus tear, and (**d**) radial meniscus tear.

Figure 7 examines the stress distribution in different tear types. In a healthy meniscus, the stress distribution is quite uniform, with a maximum stress of around 26.145 MPa in the loaded areas, indicating that the meniscus distributes loads evenly, protecting the joint. In the case of a horizontal tear, a slight stress concentration occurs at the tear site, and the maximum stress reaches 26.543 MPa. In a vertical tear, the stress concentration increases even more, reaching a maximum of 30.763 MPa, indicating that vertical tears significantly disrupt meniscal biomechanics. The radial tear exhibits the highest stress values (40.616 MPa), severely compromising the integrity of the meniscus and increasing the risk of joint damage. Overall, it is observed that the load-bearing capacity of the meniscus decreases and loads concentrate at the tear edges.

**Figure 8:**
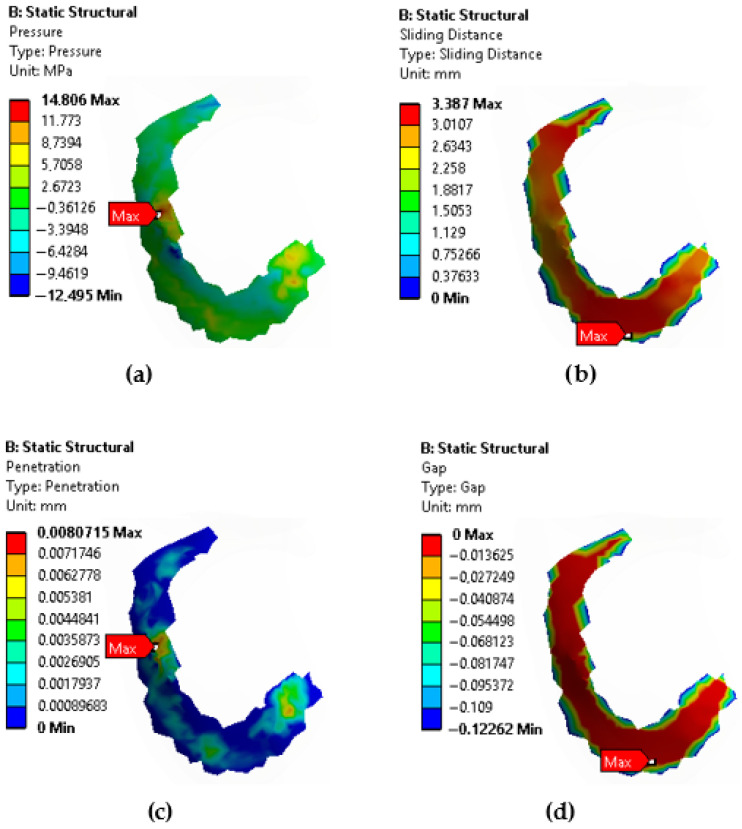
Contact surface between femoral articular cartilage and meniscus showing: (**a**) pressure, (**b**) sliding distance, (**c**) penetration, (**d**) gap.

Figure 8 presents the pressure, sliding distance, penetration, and gap values generated at the contact surface between the femoral articular cartilage and the meniscus in a healthy meniscus. Table 2 presents the values for other tear types. Wu et al. [20] investigated the effect of the knee joint alignment angle (correction angle) on the stress distribution in the meniscus and cartilage. It was shown that changes in the knee joint alignment angle significantly affected the stress distribution in the medial and lateral menisci and femoral cartilage. In particular, in the case of varus deformity, the stress on the medial meniscus increased, while the stress on the lateral meniscus decreased. In our study, we investigated the effects of different meniscus tear types (horizontal, vertical, radial) on the stress distribution within the meniscus and joint stability. While both studies focus on meniscal biomechanics, they approach the subject from different perspectives. The first study examines the direct effects of meniscal tears, while the second study examines the indirect effects of knee joint alignment on the meniscus. This allows us to better understand the complex interaction of meniscal tears and joint alignment on knee joint biomechanics. It is seen that meniscal tears affect not only the meniscal tissue but also the articular cartilage and joint stability. Moreover, it is understood that joint alignment disorders can increase the risk of tears by altering the load distribution on the meniscus.

### 3.1. Mathematical Model for Stress Estimates in Medial Meniscus Based on Mechanical Parameters

A model has been developed to understand the relationship between the stresses in the medial meniscus and the penetration, gap, pressure, and sliding distance values at the interface between the femoral cartilage and the meniscus. *S* is the stress (MPa), *P* is the pressure (Pa), *G* is the gap (mm), *P_en_* is the Penetration (mm) and *SD* is the sliding distance (mm) (Equation (1)).

Stress can be expressed as a function of the other parameters.

(1) $$S=x.P+y.G+z.P_{en}+t.SD+e$$

In this equation, the coefficients *x*, *y*, *z*, *t*, and *e* are determined using regression analysis. It can be assumed that there is a linear relationship between stress and pressure for a healthy meniscus. The rates of change and the effects of values such as penetration vary for different tear types (horizontal, vertical, radial). These data can be analyzed with a regression model using a tool such as Python (3.14.4) or MATLAB (R2026a). A Python code has been developed for this process (Appendix A).

It’s important to remember that the regression analysis in this paper only used four data points that represented the meniscal conditions under consideration: healthy, horizontal tear, longitudinal tear, and radial tear. As a result, the regression model is not designed to be a thorough or statistically sound forecasting model. Instead, it is presented as an exploratory analysis to demonstrate the initial trend in meniscus stress changes across the various tear morphologies. To create a statistically trustworthy predictive link in future research, a bigger dataset covering more tear shapes and loading conditions will be necessary.

### 3.2. Archard’s Wear Model for Contact Pressure

The present study mostly assesses stress responses derived from finite element modeling. Using well-known tribological models, wear may nevertheless be estimated computationally as well. Using Archard’s wear law, which links wear to normal contact pressure, sliding distance, the adhesive wear coefficient, and the hardness of the softer material, a reduced wear assessment was carried out in this setting.

The generalized Archard wear formulation is expressed as [30]:

(2) $$W=\int K\frac{P^{a}V^{b}}{H^{c}}dt\;$$

where W denotes cumulative wear, P is the interface pressure readily obtained from FEA, V is the sliding velocity derived from joint kinematics, H is hardness of the softer material, a, b, c and K are experimentally calibrated coefficients.

Consistent with the classical Archard formulation and customary practice in computational wear modeling of soft biological tissues, the model exponents were set to:

(3) a=b=c=1

Although nonlinear wear formulations with non-integer exponents have been proposed, the reviewed literature indicates that the vast majority of computational wear studies retain the integer-based exponents originally defined in Archard’s law. Given the absence of experimental calibration data and the aim of first-order comparative analysis, the classical Archard formulation with *a* = *b* = *c* = 1 was adopted in this study [31].

Under this assumption, the wear model simplifies to:

(4) $$W=\int K\frac{PV}{H}dt$$

The sliding distance L was determined from the known joint geometry and kinematics as:

(5) L=∫Vdt

Placing this formula into the wear equation produces the ultimate simplified wear link:

(6) $$W=K\frac{PL}{H}$$

This formulation lets one roughly estimate wear using quantities easily accessible from the finite element analysis, including contact pressure and sliding distance. Though simplified, this method helps to provide a significant comparative analysis of wear patterns under the same loading and kinematic circumstances, thus adding to the stress-based assessment given in this study. Furthermore, in order to do the wear evaluation in the TMJ, Archard’s law was adopted to account for the volume of wear of the cartilage over every loading cycle, which can be expressed as Equation (6) [32].

A simplified Archard wear model was used to provide a comparative assessment of meniscal tear effects. Contact pressure and sliding distance from the FEA were employed with classical integer exponents (*a* = *b* = *c* = 1) to estimate relative wear patterns. While quasi-static simulations limit absolute predictions, this approach offers meaningful comparative insights alongside stress analysis.

The long-term effects of different meniscal tears on joint health were compared using the Archard wear model. The use of a first-order comparative analysis based on validated Finite Element (FE) outputs is a recognized methodology in computational biomechanics, even though absolute wear volume prediction necessitates extensive tissue-specific experimental calibration [31]. The relationship between contact pressure (*P*) and sliding distance (*L*), as shown in Table 2, demonstrates the numerical consistency of this method. In particular, the Horizontal Meniscus Tear showed the greatest wear potential (W ∝ 53.13), which was 5.9% higher than the baseline for health (W ∝ 50.15). This increase in wear risk is directly correlated with the localized stress concentrations found in the FEA, indicating that horizontal lesions accelerate surface degradation through altered kinematics in addition to jeopardizing the fibrocartilage’s structural integrity. The model successfully bridges the gap between instantaneous stress distribution and the cumulative mechanical risk of degenerative joint disease by employing validated mechanical inputs within the classical Archard framework (W = KHPL), offering a reliable reference for postoperative rehabilitation planning.”

## 4. Discussion

This study highlights the critical role of meniscal tear morphology in governing knee joint biomechanics. FEA demonstrated that all medial meniscus tears increased internal stress compared with the healthy condition, with radial tears producing the most pronounced stress concentrations. This finding underscores the biomechanical importance of circumferential collagen fibers, whose disruption in radial tears severely compromises hoop stress transmission and load-sharing capacity. Despite significant increases in meniscal stress, contact pressure at the cartilage–meniscus interface remained relatively unchanged across tear types, indicating that internal stress distributions are more sensitive markers of mechanical deterioration than surface pressure alone. A modest rise in penetration values, particularly in radial tears, suggests enhanced frictional interaction at the joint interface, which may contribute to progressive cartilage damage under repetitive physiological loading. A key contribution of this study is the development of a regression-based model that reliably predicts meniscal stress from interface parameters, extending the applicability of the findings beyond descriptive biomechanics. Clinically, the combination of elevated mechanical stress and limited healing potential in avascular meniscal regions highlights radial tears as a high-risk pattern for accelerated cartilage degeneration. Overall, these results reinforce the importance of early diagnosis and meniscus-preserving treatment strategies to mitigate long-term joint degeneration.

The linear relationship between stress and pressure is supported by the regression analysis undertaken under normal meniscus conditions, which revealed that pressure is the main factor affecting stress, while other factors had little impact. This shows a linear stress–pressure dependency throughout the physiological loading range. This hypothesis is based on data and limited to the intact meniscus scenario as tear conditions clearly show more intricate, non-linear interactions.

The regression equation reveals the relationship between stress S in the medial meniscus and parameters such as pressure P, gap G, penetration $P_{en}$, and sliding distance SD, with each coefficient indicating its impact. For a healthy meniscus, stress is primarily influenced by pressure, reflecting a more linear relationship, while the effects of penetration, gap, and sliding distance remain minimal. In contrast, for meniscus tear types (e.g., horizontal, vertical, radial), the coefficients highlight how altered biomechanics at the interface amplify the roles of penetration and gap, leading to more complex stress distributions.

Though a multivariate regression model was created to characterize medial meniscus stress, statistical significance testing of single coefficients was unreliable owing to the small sample size (*n* = 4). Consequently, *p*-values exceeded the customary significance threshold (*p* > 0.05); therefore, the model should be considered as exploratory and hypothesis-generating. Robust inferential statistical validation depends on future research with bigger datasets.

Atmaca et al. [21], examined the loads on tibial cartilage according to the size and location of meniscectomy, while our study assessed the stress distributions of meniscal tears and, in particular, the effect of radial tears on meniscal tissue. Both studies address the critical role of the meniscus in knee joint biomechanics from different perspectives. The common findings of both studies indicate that the lack or impairment of the meniscus’s load-bearing and tibiofemoral contact area increasing properties can lead to increased stress on the tibial cartilage and long-term cartilage damage. Therefore, considering the biomechanical effects of meniscectomy and tear types together in the preservation or treatment of the meniscus can contribute to the development of more effective surgical and rehabilitation strategies.

Meniscal tissue is 70–90% avascular and is primarily nourished by diffusion from the synovial fluid. Only the peripheral 10–30% is supplied by the peripheral vascular network, which originates from branches of the medial and lateral genicular arteries [33]. Meniscal tears can also be classified into three zones: the outer vascular zone (red-red zone), the inner avascular zone (white-white zone), and the intermediate zone (red-white zone) [34]. Given the meniscal blood supply pattern, the peripheral vascular area of the meniscus has a high capacity for healing, while the more central avascular zone has no healing potential. In studies that examined patients who underwent arthroscopic meniscal repair, a positive correlation was found between the proximity of the tear to the peripheral vascular network and healing [35]. Since the tear reaches the red-red zone, there is a reality that excessive stress distribution on the torn surfaces can affect healing and lead to non-union of meniscus in surgically or not surgically treated patients.

Finite element simulations showed clear variations in stress and strain patterns between damaged and undamaged meniscal tissue models. Statistical analyses of important biomechanical parameters, including peak von Mises stress and maximum principal strain, showed notable variation across the simulated cases. These statistically significant changes show that the presence of meniscal injury causes mechanically relevant changes in load distribution and local stress concentration patterns. Supporting the interpretation of computational model results with statistical analysis is in line with accepted methods in computational biomechanics for evaluating model credibility and mechanical relevance [36].

Additionally, dynamic numerical modeling in FEA—that is, time-dependent assessment of interaction factors and contact circumstances—is performed during motion. While a quasi-static formulation is employed, the current model incorporates dynamic numerical behavior via kinematically driven alterations in contact pressure, penetration, gap, and sliding distance. Dynamic numerical simulation ideas described in the literature, wherein the temporal evolution of contact variables is employed to reflect actual system behavior under motion [37].

The stress values obtained from the present finite element simulations were compared with previously published studies in the literature to assess the plausibility of the results. Previous research has reported that meniscal tears significantly increase stress concentrations within the meniscus and alter load distribution in the knee joint. Our findings demonstrate a similar trend, where all tear configurations produced higher stress values than the healthy meniscus condition. The magnitude and pattern of stress increase observed in this study are consistent with earlier finite element investigations, confirming that the model provides biomechanically reasonable results and supporting the validity of the present simulation approach [38].

This study’s finite element model has a number of simplifications. To provide a standardized geometry for comparative analysis, an educational anatomical knee model was utilized in place of patient-specific medical imaging data. Although actual meniscal tissue behaves anisotropically, the meniscus was modeled as a homogeneous and isotropic material to minimize computational complexity. For comparison, a single static load of 350 N was used as a reference physiological loading condition. Furthermore, rather than offering a thorough statistical model, the regression analysis was based on four data points and was only meant to show initial trends.

## 5. Conclusions

This study quantitatively revealed the effects of medial meniscus tears on knee joint biomechanics using Finite Element Analysis (FEA). The numerical data obtained confirm that the stress value, which is 26.145 MPa in a healthy meniscus, increases in all tear types, and that this value reaches 40.616 MPa in radial tears, causing the highest stress concentration. Simulation results show that the sliding distance increases with tear progression, while the gap values remain constant at approximately −0.122 mm in all scenarios, exhibiting consistent tissue penetration and contact stability under load. The strong correlation (R^2^ > 0.9) offered by the developed mathematical model between stress and interface parameters proves that the decrease in the load-bearing capacity of the meniscus can be numerically predicted. Although not including biological variables such as age, gender, or vascular structure, the presented biomechanical findings demonstrate that radial tears are the type of tear that increases the mechanical load on cartilage the most, and these data provide a scientific basis for the mechanical optimization of artificial meniscus designs and rehabilitation strategies.

## Figures and Tables

**Table 2:** Stresses in medial menisci.

Type	Stress Distributions for Medial Meniscus				
	Stress (MPa)	Pressure (MPa)	Gap (mm)	Penetration (mm)	Sliding Distance (mm)
Healthy Meniscus	26.145	14.806	−0.12262	0.0080715	3.387
Horizontal Meniscus Tear	26.543	15.236	−0.12242	0.00806652	3.487
Longitudinal (vertical) Meniscus Tear	30.763	12.107	−0.12262	0.006995	3.4686
Radial Meniscus Tear	40.616	15.078	−0.12255	0.00831	3.467

## Data Availability

All data generated or analyzed during this study are included in this article. Further enquiries can be directed to the corresponding author.

## Appendix A

import numpy as np

from sklearn.linear_model import LinearRegression

# Data

pressure = np.array([14.806, 15.236, 12.107, 15.078])

gap = np.array([−0.12262, −0.12242, −0.12262, −0.12255])

penetration = np.array([0.0080715, 0.00806652, 0.006995, 0.00831])

sliding_distance = np.array([3.387, 3.487, 3.4686, 3.467])

stress = np.array([26.145, 26.543, 30.763, 40.616]) # lowercase consistent

# Independent variables

X = np.column_stack((pressure, gap, penetration, sliding_distance)) # uppercase consistent

y = stress # lowercase matches the data variable

# Linear regression model

model = LinearRegression().fit(X, y)

# Coefficients and intercept

print(“Coefficients (pressure, gap, penetration, sliding_distance):”, model.coef_)

print(“Intercept:”, model.intercept_)
